# Effect of awake prone positioning on tracheal intubation rates in patients with COVID-19: A meta-analysis

**DOI:** 10.1016/j.heliyon.2023.e19633

**Published:** 2023-09-01

**Authors:** Dan Wen, Xiuru Yang, Zhenghua Liang, Fenglin Yan, Haiyan He, Li Wan

**Affiliations:** a Mianyang Central Hospital, School of Medicine, University of Electronic Science and Technology of China, Mianyang, Sichuan Province, China; b Department of Nursing, Mianyang Central Hospital, School of Medicine, University of Electronic Science and Technology of China, Mianyang, Sichuan Province, China

**Keywords:** Awake prone position, COVID-19, Tracheal intubation, Meta-analysis

## Abstract

**Purpose:**

We investigated the effect of awake prone positioning on endotracheal intubation rates in spontaneously breathing patients with COVID-19 not undergoing endotracheal intubation.

**Methods:**

We searched the CINAHL, Cochrane Library, PUBMED, MEDLINE, and Web of Science databases until December 31, 2022. Prospective randomized controlled, cohort, and case-control studies were included. A meta-analysis was performed on the primary outcome measure, tracheal intubation rates, following the Preferred Reporting Items for Systematic Reviews and Meta-Analyses (PRISMA) guidelines.

**Results:**

Ten studies with a total of 2641 patients were included. The tracheal intubation rate in the awake prone position was 34% (95%CI: 0.59–1.10; *P* = 0.18; *I*^2^ = 55%), showing a non-significant benefit. Mortality was lower in prone-positioned than in supine-positioned patients (odds ratio: 0.75; 95% CI: 0.61–0.93; *P* = 0.007; *I*^2^ = 46%), prone positioning significantly improved the PaO2/FiO2 ratio (mean difference −29.17; 95%CI: −50.91 to −7.43; *P* = 0.009; *I*^2^ = 44%).

**Conclusions:**

Prone positioning can improve the PaO2/FIO2 ratio in patients with COVID-19 but we found no significant effect on tracheal intubation rates. Awake prone positioning seems to be associated with lower mortality, however, and may thus be a beneficial and effective intervention for patients with COVID-19. The optimal timing, duration, and target population need to be determined in future studies.

## 1 Introduction

The coronavirus 2019 (COVID-19) disease, caused by the severe acute respiratory syndrome coronavirus 2 (SARS-CoV-2), has resulted in unprecedented morbidity and mortality [1,2]. The COVID-19 pandemic has led to the hospitalization of approximately 270,000 people in intensive care units (ICUs) between May 1, 2020 and March 10, 2022 in Europe alone [3]. The unprecedented surge in the number of critically ill patients is exerting immense pressure on ICU capacity globally [4,5].

The prone position has been used since the 1970s to administer invasive mechanical ventilation to patients with acute respiratory distress syndrome (ARDS) [6]. Studies have shown that prone positioning increases lung volume, reduces lung tension due to changes in pleural pressure and pleural space distribution, facilitates ventilation/perfusion matching [7]. Randomized clinical trials found that the prone position is associated with a lower risk of death in patients with moderate-severe ARDS receiving invasive mechanical ventilation (95% CI: 0.56–0.99) [8]. The longer patients receive prone treatment, the greater the benefit [[9], [10], [11]], which improves oxygenation overall and is associated with reduced mortality in patients with mechanical ventilation [12]. Prior to the COVID-19 the pandemic, the awake prone positioning had been used to reduce intubation rates and mortality in patients with acute respiratory failure and acute respiratory distress syndrome.

The majority of patients with COVID-19 have been reported to develop acute respiratory distress syndrome. Fazzini [7], in a single-center study, assessed the effects of awake prone positioning during spontaneous respiration on oxygenation and clinical outcomes in patients with the novel coronavirus. The authors found that prone positioning was associated with significant improvements in oxygenation, reduced ICU admissions, reduced tracheal intubation, and reduced length of ICU stay. A recent study published in JAMA explored the effects of awake prone positioning on endotracheal intubation in patients with COVID-19 with acute respiratory failure, however, and found that it did not significantly reduce tracheal intubation within 30 days, compared to conventional care without prone positioning [13].

The effectiveness of awake prone positioning in reducing intubation rates and mortality remains unclear [14,15], and the available evidence on patient tolerance, the required timing, and optimal duration is inconsistent [16]. Given the insufficiency of evidence, it is imperative to assess the efficacy of awake prone positioning as an adjunctive therapy for patients with COVID-19 hypoxemia. Here we therefore conducted a meta-analysis to systematically evaluate the efficacy and safety of awake prone positioning in patients with COVID-19, with the aim to provide a theoretical basis as well as evidence for clinical treatment. We believe these findings may also help patients with acute hypoxemia and respiratory failure to perform awake prone positions.

## 2 Methods

The Preferred Reporting Items for Systematic Reviews and Meta-Analyses (PRISMA) guidelines [17] were followed in the review process and analyses. The study protocol was registered in PROSPERO (CRD42023402513).

### 2.1 Search strategy

The CINAHL, Cochrane Library, PUBMED, MEDLINE, and Web of Science databases were searched from the date of creation to December 31, 2022. The search was performed using combination keywords of COVID 19 OR Covid-19 OR SARS Cov 19 OR SARS-COV 2019 OR SARS-COV-19 OR corona virus disease OR corona virus 19 disease AND prone positioning OR proning OR proning position. Search results were restricted to studies with adult participants published in English.

### 2.2 Inclusion and exclusion criteria

We applied the following inclusion criteria: type of study (randomized or quasi-randomized controlled trials, cohort studies, or case-control studies); type of participants (those confirmed with reverse transcription polymerase chain reaction (RT-PCR) tests or imaging findings showing evidence of COVID-19 requiring supplemental oxygen or non-invasive CPAP); type of intervention (patients were instructed to stay in the prone position, based on the protocol of each study, for at least 30–60 min and then return to the supine position); type of outcome (primary outcome: rate of endotracheal intubation; secondary outcome: mortality and PaO2/FiO2 ratio). Studies were excluded when they were published in non-English languages or as conference abstracts, case reports, or letters. Studies on pregnant women or patients with contraindications to prone positioning, such as skeletal fractures, were also excluded.

### 2.3 Study selection and data extraction

We removed duplicate articles from the search results using EndNote software (version 9.3). The literature was then screened by two researchers (ZH and HH) according to the inclusion and exclusion criteria outlined above. Disagreements were discussed with a third researcher (XY) and a joint decision was made to be included in the study. The extracted data included the first author, publication time, total sample size, age, intervention measures, and outcome indicators.

### 2.4 Quality evaluation

We assessed the quality of observational cohort studies using the Newcastle-Ottawa Scale (NOS). A score of 0–4 indicated low-quality literature (grade C), 5–6 medium-quality literature (grade B), and 7–9 high-quality literature (grade A). The quality of randomized controlled trials was assessed using the bias risk assessment tools recommended in the Cochrane Review Manual 5.1.0. If the standards were fully met, a study was awarded the quality grade A. If partially satisfied, we awarded a grade B, and a grade C was awarded when none of the standards were met.

### 2.5 Statistical analysis

The statistical analysis was performed using Review Manager 5.4 (RevMan 5.4.1) provided by the Cochrane Collaboration (Oxford, UK). We reported dichotomous outcomes using odds ratio (OR) and continuous outcomes using mean difference (MD). The combined effect was assessed by determining the OR and MD for 95% confidence intervals (CIs). Heterogeneity among the included studies was assessed by Q and *I*^2^ tests. A random-effects model was adopted for the analysis considering variation across the included studies. The stability of the results was assessed with a sensitivity analysis, by eliminating one study at a time.

## 3 Results

### 3.1 Study selection

**Fig. 1:**
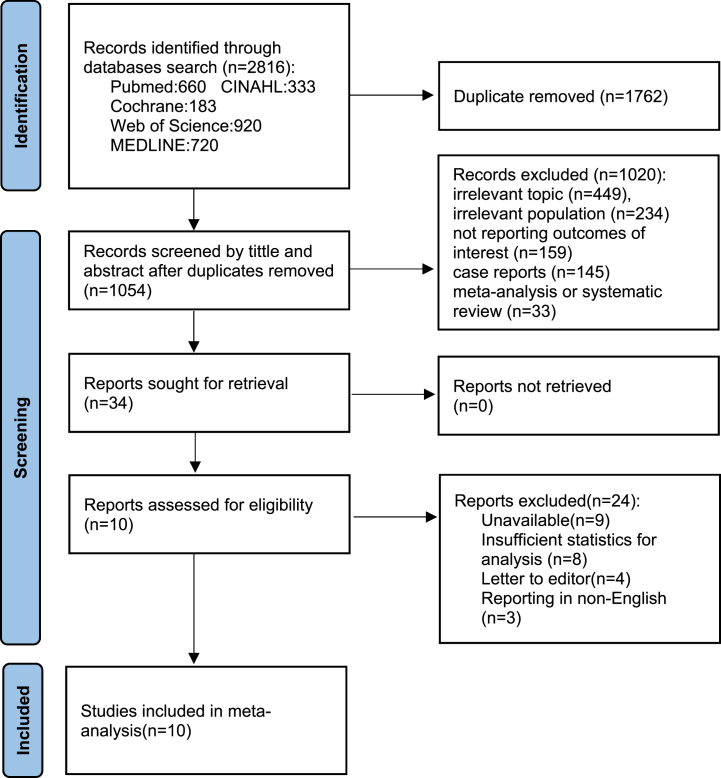
PRISMA flowchart.

The PRISMA flowchart (Fig. 1) shows that 2816 articles were originally retrieved from the included databases; 1054 were retained after removing duplicates. During the first screening of titles and abstracts, 1020 articles were excluded for the following reasons: irrelevant topic (449 articles), irrelevant population (234 articles), not reporting outcomes of interest (159 articles), case report (145 articles), or meta-analysis or systematic review (33 articles). Ten articles that met all conditions were identified through a full-text screening and included in the final analysis [5,13,15,[18], [19], [20], [21], [22], [23], [24]].

### 3.2 Study characteristics and quality

The main features extracted from the studies included in our analysis are listed in Table 1. All studies were published after 2020 and included a total of 2356 patients, with sample sizes ranging from 56 to 1121 participants per study. In the quality assessment, eight studies were given a grade B [5,13,15,19,[21], [22], [23], [24]], and two a grade C [18,20]. Details on the assessment for each study are provided in Table 2.

**Table 1:** Characteristics and quality of included studies.

Study	Country	Study Design	Clinical setting	N	Oxygen delivery mode	Duration of PP	Primary outcome	Study quality grading
Alhazzani	Canada Kuwait Saudi Arabia USA	Prospective	ICU	400	HFNC CPAP	8–10 h/day	Endotracheal intubation within 30 days	B
Coppo	Italy	Prospective	ICU ED Ward	56	CPAP Reservoir mask Venturi mask	At least 3 h a day	PaO2/FiO2	C
Ehrmann	Mexico France USA Spain Ireland Canada	Prospective	ICU HDU ED Ward	1126	HFNO	As long and as frequently as possible each day	Intubation or death within 28 days of enrolment	B
Ferrando	Spain and Andorra	Prospective	ICU	199	HFNO	16 h/day during 3 consecutive day	PaO2/FiO2	B
Jagan	USA	Retrospective	ICU	105	Not mentioned	One hour at a time, five times a day	Intubation during the patient's hospital stay	C
Jayakumar	India	Prospective	ICU	60	Face mask HFNC NIV	At least 6 h a day	The proportion of patients adhering to the protocol	B
Padrao	Brazil	Retrospective	ED	166	face mask, HFNO NIV	Between 30 min and 4 h	Orotracheal intubation up to 15 days after inclusion	B
Rosen	Sweden	Prospective	ICU	75	HFNC NIV	At least 16 h day	Intubation within 30 days after enrollment.	B
Tonelli	Italy	Prospective	ICU	114	HFNC CPAP NIV	3 h/day, 1–4 times a day	Tracheal intubation rate	B
Zang	China	Prospective	Not reported	60	NC HFNC NIV	Median:9 h (8–22)	Tracheal intubation rate	B

**Table 2:** Quality and risk of bias assessment using the Newcastle-Ottawa Scale (NOS) for observational studies.

Study ID	Selection				Comparability	Outcome		Total (7*)
	Representativeness of the exposed cohort (*)	Selection of non-exposed cohort (*)	Ascertainment of exposure (*)	Demonstration that outcome of interest was not present at start of study (*)	Comparability of cohorts (*)	Assessment of outcome (*)	Adequacy of follow up (*)	
Coppo 2020			*	*		*	*	4
Ferrando 2020		*	*	*	*	*	*	6
Jagan2020			*	*		*	*	4
Jayakumar2021		*	*	*	*	*	*	6
Padrao 2020		*	*	*	*	*	*	6
Tonelli 2020		*	*	*	*	*	*	6
Zhang 2020		*	*	*	*	*	*	6

### 3.3 Primary outcome: intubation

**Fig. 2:**
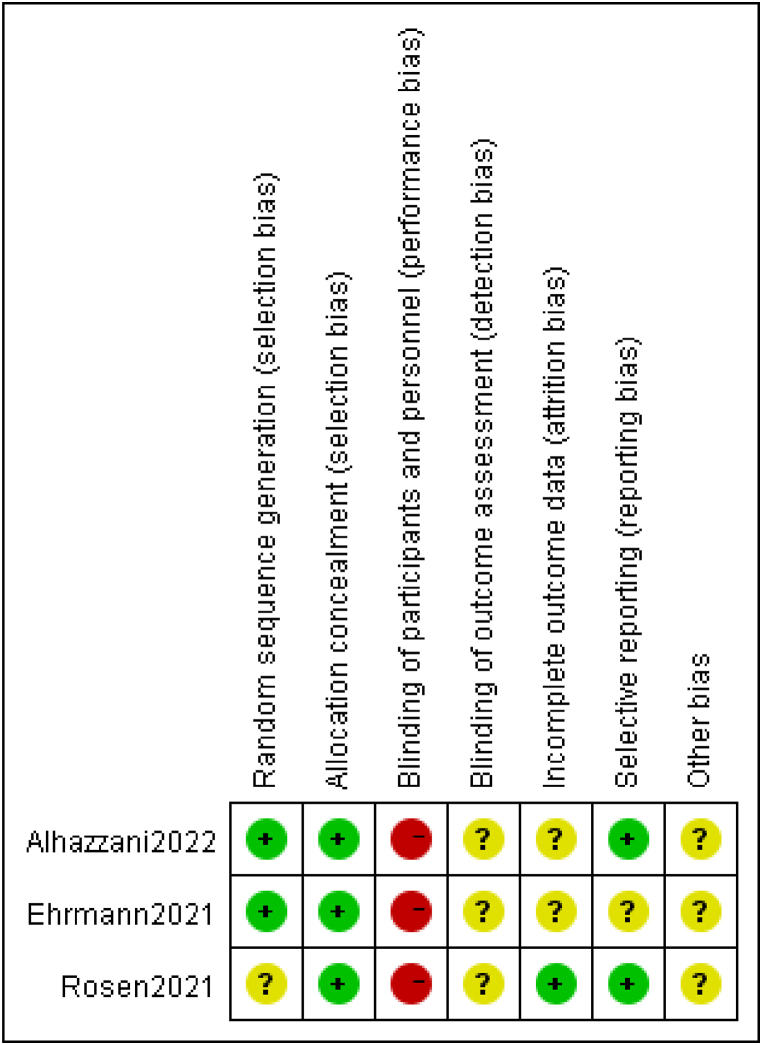
Risk for bias assessment using the Cochrane collaboration tool for randomized trial.

The tracheal intubation rate was 33%, derived from a total of 1095 patients with COVID-19 treated in the awake prone position. The OR for intubation in the awake prone position, compared with supine-positioned control patients, was 0.80 (95% CI: 0.58–1.11; *P* = 0.19; *I*^2^ = 50%), showing a non-significant benefit (Fig. 2).

### 3.4 Secondary outcomes

**Fig. 3:**
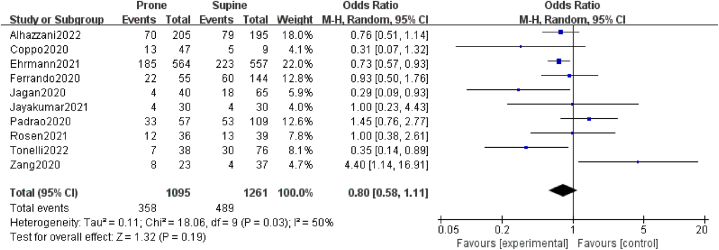
Forest plot the effects of prone position towards intubation.

The pooled mortality of patients placed in the prone position (reported in eight studies) was 19.6%. The OR for mortality was 0.77 (95% CI: 0.62–0.95; *P* = 0.01; *I*^2^ = 51%), indicating a statistically significant improvement in prone-positioned compared to supine-positioned patients (Fig. 3).

**Fig. 4:**
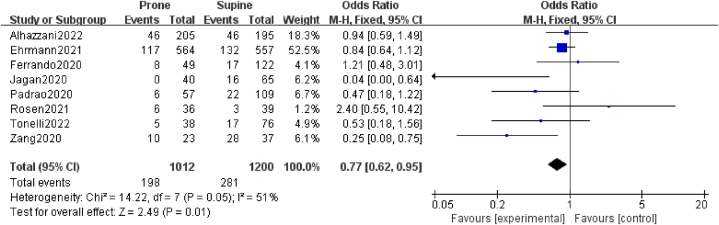
Forest plot the effects of prone position towards mortality.

**Fig. 5:**
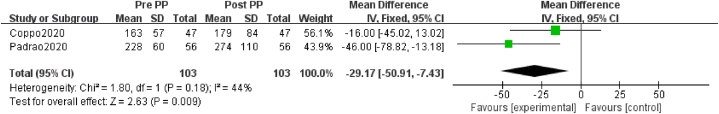
Forest plot the effects of prone position towards PaO2/FiO2.

The mean differences (MD) in PaO2/FiO2 ratio before and after awake prone positioning was −29.17 (95%CI: −50.91 to −7.43; *P* = 0.009; *I*^2^ = 44%), based on the outcomes of two studies that reported these details. Prone positioning thus significantly improved PaO2/FiO2 (Fig. 4) (see Fig. 5).

## 4 Discussion

We pooled the results of 10 prospective, retrospective, and randomized controlled trials involving 2356 non-intubated patients with COVID-19, including 1095 treated in the prone position and 1261 in the non-prone position.

Our results demonstrate that the role of awake prone positioning in reducing tracheal intubation is still unclear. Four studies included in this meta-analysis reported that awake prone positioning reduced the incidence of treatment failure, thereby leading to a lower intubation rate. In contrast, six studies found no reduction in the risk of intubation. The rate of endotracheal intubation in the awake prone position was 33% across all studies included in our analysis, which is higher than the 28% reported in an earlier meta-analysis by Cardona [25]. When awake patients are in a prone position, changes in pleural pressure and spatial distribution of the pleura throughout the lung area promote more uniform ventilation. Prone positioning may reduce the risk of endotracheal intubation in patients with PaO2/FiO2 values above 150 mmHg and in those receiving high-flow oxygen, but it may not benefit patients with more severe disease [13]. Most studies included in this analysis were conducted in the ICU, and the severity of hypoxia varied greatly across patients, leading to a high rate of tracheal intubation. The following reasons can be put forward to explain this finding. Prone positioning may have to be used in conjunction with other oxygen delivery methods associated with reducing intubation rates [26]. The duration of prone positioning may also be important, as the PROSEVA trial showed a statistically significant benefit among intubated patients who were prone-positioned for an average of 17 h [27]. It is also possible that only a small percentage of patients, whose characteristics have not been determined, benefit from awake prone positioning.

Prone positioning can improve chest wall and lung compliance in awake patients, reduce lung injury, and facilitate secretion discharge under gravity, reduce heart pressure on the lung, change diaphragm movement and reducing mortality. However, there may be a risk of delayed intubation when patients with COVID-19 are placed in the awake prone position, which is associated with increased mortality in critically ill patients [28,29]. It is worth noting that two reports included in this analysis found that awake prone positioning can reduce mortality in patients with COVID-19, while the remaining six studies indicated no such difference between patient groups. However, our meta-analysis results show that prone positioning does indeed lead to lower mortality rates; the discrepancy may stem from differences in implementation protocols for prone positioning across studies and short mortality monitoring times. Mortality indicators in the referenced studies included 15 and 28 days, but there was a lack of long-term (≥3 months) mortality data. The relationship between awake prone positioning and long-term mortality in patients with COVID-19 therefore needs further study.

Two studies showed significant improvements in the PaO2/FiO2 ratio after prone positioning. By increasing the PaO2/FiO2 ratio resulting in a lower requirement for oxygen concentration, the lung injury caused by prolonged high oxygen concentration is reduced. Typical ARDS is associated with reduced lung compliance and severe hypoxemia. Lung injury associated with COVID-19 can be managed using the lung protective ventilation strategies that are also employed for ARDS [30]. The mechanism by which prone positioning improves oxygenation in ARDS is, however, complex. In the prone position, lung density is redistributed, which relieves the effects of heart and mediastinal compression and allows the collapsed alveoli in the dorsal lung region to re-expand. Changes in the pleural pressure gradient and gravity as well as redistribution of cross-lung pressure then improves lung ventilation and blood perfusion. With the change in pleural pressure and pleural space distribution throughout the lung area, lung stretch and tension decrease in the prone position, which facilitates more uniform ventilation [31].

The time awake patients need to maintain the prone position for effects to occur has not been determined, and no clear standards for when patients should move out of the position have been proposed. Discomfort is the main cause for short durations or interruptions of prone positioning, and since patient cooperation is required, patient tolerance and compliance are crucial. A study by Elharrar [32] in 24 patients with COVID-19 requiring oxygen found that only 63% (15/24) were able to undergo prone ventilation for more than 3 h. Of these 15 patients, 40% (6/15) exhibited improved oxygenation during ventilation, while those undergoing ventilation for less than 3 h showed no significant improvement in oxygenation.

Studies have recommended to include awake prone-position ventilation in the treatment of COVID-19, but this approach is still deficient in many ways [33]. First, patient cooperation is needed, and many patients cannot tolerate the treatment, which limits its clinical application. Second, there is no unified standard for when to initiate awake prone positioning, and evidence on the required treatment time is also lacking [34]. ARDS guidelines recommend prone ventilation for at least 16 h per day for patients with a PaO2/FiO2 value below 150 mmHg [35]. However, most available studies on awake prone ventilation had patients maintain the prone position for only 2–3 h a day [36]. Therefore, further studies need to determine whether short-time prone ventilation can also reduce mortality. Patients with mild to moderate ARDS may exhibit varying degrees of dorsal alveolar collapse, ventilation/perfusion mismatch, and other pathological factors. Early prone positioning while awake can also be beneficial for these pathologies. Current research on prone positioning for awake patients primarily focuses on COVID-19, and further investigation is necessary to determine its effectiveness for non-COVID-19 induced ARDS patients.

### 4.1 Limitations

This study has some limitations. First, only three reports included in this study were randomized controlled trials, while all others were retrospective cohort studies lacking high-quality evidence. Second, since these retrospective studies were not randomized, the characteristics of patients included in the two evaluated groups may have differed. In addition, differences in the duration of prone positioning and the number of days prone positioning was applied across studies may have led to differences in the therapeutic effect as well. Third, many studies report results over a predefined period of time rather than over the patient's entire hospital stay. This limits our understanding of the effects of awake prone positioning on the patient's overall disease course. Fourth and last, many of the included studies did not report data on key indicators of patient respiratory status, such as the PaO2/FiO2 ratio, ROX index, or chest X-ray or computed tomography results. This prevented us from comprehensively and accurately describing the included patient population.

## 5 Conclusions

In this systematic review and meta-analysis of 10 carefully selected studies, we compared prone versus supine positioning in awake patients with COVID-19. Our analysis shows that prone positioning can improve the PaO2/FiO2 ratio but has no significant effect on tracheal intubation rates. Awake prone positioning seems to be associated with lower mortality, however, and we thus recommend this approach for awake patients with COVID-19, as a potentially beneficial and effective intervention as well as to address shortages of ventilators such as those seen earlier during the COVID-19 pandemic. The optimal timing, duration, target population, intervention methods, and implementation protocols for prone positioning still need to be more rigorously explored in larger, well-designed studies and should be the focus of future research.

## Funding

This work was supported by the 10.13039/100020130Primary Health Development Research Center of Sichuan Province Program [grant numbers SWFZ22-C-96].

## Author contribution statement

All authors listed have significantly contributed to the development a>

## Data availability statement

Data included in article/supp. material/referenced in article.

## Additional information

No additional information is available for this paper.

## Declaration of competing interest

The authors declare that they have no known competing financial interests or personal relationships that could have appeared to influence the work reported in this paper.
